# Genetic data from the extinct giant rat from Tenerife (Canary Islands) points to a recent divergence from mainland relatives

**DOI:** 10.1098/rsbl.2021.0533

**Published:** 2021-12-22

**Authors:** Pere Renom, Toni de-Dios, Sergi Civit, Laia Llovera, Alejandro Sánchez-Gracia, Esther Lizano, Juan Carlos Rando, Tomàs Marquès-Bonet, Gael J. Kergoat, Isaac Casanovas-Vilar, Carles Lalueza-Fox

**Affiliations:** ^1^ Institute of Evolutionary Biology (CSIC-Universitat Pompeu Fabra), Barcelona 08003, Spain; ^2^ University of Tartu, Institute of Genomics, Estonian Biocentre, Tartu 51010, Estonia; ^3^ Departament of Genetics, Microbiology and Statistics-Institut de Recerca de la Biodiversitat (IRBio), Universitat de Barcelona, Barcelona 08028, Spain; ^4^ Institut Català de Paleontologia Miquel Crusafont, Universitat Autònoma de Barcelona, Cerdanyola del Vallès, Barcelona 08193, Spain; ^5^ Departamento de Biología Animal, Edafología y Geología, Universidad de La Laguna, La Laguna 38206, Tenerife, Spain; ^6^ Catalan Institution of Research and Advanced Studies (ICREA), Barcelona 08010, Spain; ^7^ CNAG-CRG, Centre for Genomic Regulation, Barcelona Institute of Science and Technology (BIST), Barcelona 08036, Spain; ^8^ CBGP, INRAE, IRD, CIRAD, Institut Agro, Univ Montpellier, Montpellier, France

**Keywords:** ancient DNA, body mass, gigantism, insular evolution, molecular phylogeny, rodents

## Abstract

Evolution of vertebrate endemics in oceanic islands follows a predictable pattern, known as the island rule, according to which gigantism arises in originally small-sized species and dwarfism in large ones. Species of extinct insular giant rodents are known from all over the world. In the Canary Islands, two examples of giant rats, †*Canariomys bravoi* and †*Canariomys tamarani*, endemic to Tenerife and Gran Canaria, respectively, disappeared soon after human settlement. The highly derived morphological features of these insular endemic rodents hamper the reconstruction of their evolutionary histories. We have retrieved partial nuclear and mitochondrial data from †*C. bravoi* and used this information to explore its evolutionary affinities. The resulting dated phylogeny confidently places †*C. bravoi* within the African grass rat clade (*Arvicanthis niloticus*). The estimated divergence time, 650 000 years ago (95% higher posterior densities: 373 000–944 000), points toward an island colonization during the Günz–Mindel interglacial stage. †*Canariomys bravoi* ancestors would have reached the island via passive rafting and then underwent a yearly increase of mean body mass calculated between 0.0015 g and 0.0023 g; this corresponds to fast evolutionary rates (in darwins (d), ranging from 7.09 d to 2.78 d) that are well above those observed for non-insular mammals.

## 1. Introduction

**Figure 1.**
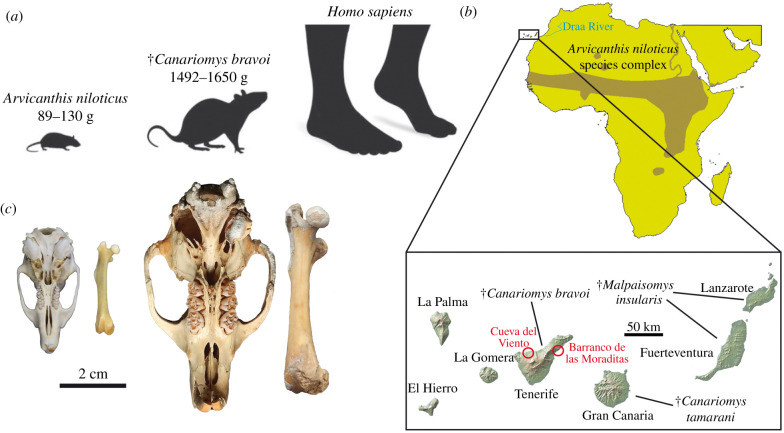
(*a*) Outline drawings and body mass for *A. niloticus* and †*C. bravoi*, along with a human reference. (*b*) Current distribution of *A. niloticus* in Africa (data from IUCN, 2008). The inset shows the Canary Islands and the distribution of extinct endemic rodents. Sampling sites are indicated for Tenerife. (*c*) Size differences between a typical *A. niloticus* representative (left) and †*C. bravoi* (right) as illustrated by their crania and femora (specimens are curated in IMEDEA and DZUL collections with numbers 12758 (*A. niloticus*) and 3199 (†*C. bravoi*), respectively). The latter has not been subjected to DNA analysis.

The Canary Islands are located northwest off the coast of Africa, with their nearest island (Fuerteventura) being only separated from the continent by about 100 km (figure 1*b*). Although this volcanic archipelago was never connected to the mainland by any land bridge or island chain, colonization of terrestrial organisms from the mainland was favoured by dominant oceanic currents. This archipelago offers a unique opportunity to study the colonization and diversification of multiple groups of organisms, such as birds, reptiles or small mammals [1]. Among the latter, there are three known striking examples of gigantism: the lava mouse of Fuerteventura and Lanzarote (†*Malpaisomys insularis*) and the extinct giant rats of Tenerife and Gran Canaria (†*Canariomys bravoi* and †*Canariomys tamarani*, respectively).

The Tenerife giant rat was described by Crusafont-Pairó & Petter [2] after the discovery of numerous specimens in Quaternary sites. Subsequent studies explored its diet, ecology, body mass and extinction causes, and also tentatively assessed its phylogenetic affinities based on dental traits (e.g. [3,4]). †*Canariomys bravoi* shows a set of traits characteristic of insular rodents, including gigantism, a robust skeleton and high-crowned teeth (figure 1*c*). It became extinct after the fourth century BCE, likely in relation to the arrival of Canarian indigenous people [4]. †*Canariomys tamarani* also became extinct soon after the arrival of the first settlers while †*M. insularis* survived until the beginning of the fourteenth century, when Europeans reached the archipelago [5]. Ancient mitochondrial DNA (mtDNA) from †*M. insularis*, showed close affinities to the extant genus *Mus* and pointed to a 6.9 Ma divergence date (genetic data were obtained by means of the traditional polymerase chain reaction method).

Hot and humid thermal conditions hamper the retrieval of ancient genetic data [6]. Without this information, it is difficult to unravel the affinities of highly modified extinct species such as †*C. bravoi* to their mainland smaller relatives. Here, we managed to retrieve partial nuclear and mtDNA data from two †*C. bravoi* specimens; we subsequently used this information to provide divergence age estimates and phylogenetic relationships for this lineage and determine the rate of increase in body size of this insular rodent.

## 2. Material and methods

### (a) The samples

We performed DNA extraction from 12 mandibles: two from Barranco Moraditas and 10 more from Cueva del Viento (figure 1*b*). Specimens used for extractions were deposited in the Vertebrate collection (DZUL) of Departamento de Biología Animal, Edafología y Geología de la Universidad de La Laguna (Tenerife) with the following inventory numbers: CB-1 (DZUL 3200); CB-2 (DZUL 3201); CB-3 (DZUL 3202); CB-4 (DZUL 3203); CB-5 (DZUL 3204); CB-6 (DZUL 3205); CB-7 (DZUL 3206); CB-8 (DZUL 3207); CB-9 (DZUL 3208); CB-10 (DZUL 3209); CB-11 (DZUL 3210) and CB-12 (DZUL 3211).

Cueva del Viento site is a 17 km-long system of volcanic lava tubes formed 0.17–0.13 Ma [7] and situated in the north side of Tenerife at 700 m above sea level. The animals went into the cave through a small pit fall that acted like a trap. Bones were found in connection, showing the absence of transport after deposition. Previous calibrated radiocarbon ages of †*C. bravoi* samples from this site are between 17 300 and 2150 cal BP [4,8]. The samples from Barranco de las Moraditas were recovered from a small cave infilling in basaltic materials of Quaternary age at the east of Tenerife [7]. The median age reported for another †*C. bravoi* sample from this site is 2310 cal BP [4].

### (b) DNA extraction, mitochondrial DNA capture and library preparation

All DNA extraction and initial library preparation steps were performed in a dedicated clean laboratory, physically isolated from the laboratory used for post-PCR analyses; no previous work on extinct or extant rodents was ever conducted in our laboratory. Strict protocols were followed to minimize the amount of human DNA in the ancient DNA laboratory, including wearing a full-body suit, sleeves, shoe covers, clean shoes, facemask, hair net and double gloving.

First, teeth samples were UV irradiated (245 nm) for 10 min and the outermost surface of the teeth was scraped off with a drill engraving cutter, followed by another UV irradiation in order to exclude the surface DNA contamination. Second, approximately 30 mg of tooth cementum was obtained by drilling at low speed (5000 r.p.m.) with a new engraving cutter.

DNA extraction from teeth powder was performed following the method of Dabney *et al*. [9]. The teeth powder samples, including an extraction blank, were added to 1 ml of extraction buffer (final concentrations: 0.45 M EDTA, 0.25 mg ml^−1^ proteinase K, pH 8.0), resuspended by vortexing and incubated at 37°C overnight on rotation. The remaining tooth powder was then pelleted by centrifugation in a bench-top centrifuge at maximum speed (16 100*g*). The supernatant was added to 10 ml of binding buffer (final concentrations: 5 M guanidine hydrochloride, 40% (vol/vol) isopropanol, 0.05% Tween-20 and 90 mM sodium acetate (pH 5.2)) and purified on a High Pure Extender column (Roche). DNA samples were eluted with 45 µl of EDTA TE buffer (pH 8.0). However, 10 samples failed to yield quantifiable DNA after extraction and only two from Cueva del Viento (CB-4 and CB-10) were further selected for library building.

A total of 35 µl of each DNA extract was used for library preparation in three sequential reactions: end-repair, adapter ligation, and nick fill-in; following the BEST protocol [10]. DNA extract from CB-4 was used for DNA-library preparation prior to Illumina sequencing; the resulting library was amplified by PCR with two uniquely barcoded primers and used for shotgun sequencing. Both libraries were purified with a 1× AMPure clean (Beckman Coulter) and eluted in 25 µl of low EDTA TE buffer (pH 8.0). Library size and concentration were determined with the Agilent DNA 7500 Kit on the 2100 BioAnalyzer instrument. The DNA libraries were sequenced using HiSeq400 of Illumina platform (Illumina, USA) in Macrogen, Inc. biotechnology company.

After library preparation, sample CB-10 was enriched for mtDNA sequences with the use of commercially biotinylated probes for mouse mtDNA (MYbaits). Prior to hybridization, the DNA library (approx. 500 ng) was brought to 7 µl using a Speedvak concentrator. Two consecutive hybridizations were conducted with the myBaits Capture Kit (Arbor Biosciences) according to the manufacturer's manual v. 4.01. The hybridization reaction was carried out at 65°C for 24 h in a final volume of 30 µl. Captured targets were recovered with Dynabeads MyOne Streptavidin C1 magnetic beads (Invitrogen), followed by bead–bait binding and washing according to the manufacturer's recommendations. After the first round of enrichment, post captured amplification was performed using PCR primers IS5 and IS6. All of the captured material was concentrated to 7 µl and used for the second round of hybridization. The second hybridization was performed under the same conditions and the final captured pool was amplified with P5 and P7 indexed primers compatible for Illumina sequencing [11]. Sample CB-10 was radiocarbon dated to 2800 ± 30 years BP (Beta-598676).

### (c) Phylogenetic analysis

All resulting DNA reads from samples CB-4 and CB-10 were mapped (edit distance equal to 0.0001) to *Mus musculus* (MN964117.1), *Rattus rattus* (NC_012374.1) and *Arvicanthis niloticus* (CM022273.1) mtDNA genomes. Mapped reads were subsequently blasted and only reads specific to rodent mitogenomes were retained. Additionally, DNA reads from CB-4 were mapped against the *A. niloticus* nuclear genome (NCBI:txid61156) with standard aDNA edit distance (0.01).

The authenticity of the generated sequences was confirmed with the observation of the typical post-mortem ancient DNA damage at the end of the DNA reads (electronic supplementary material, figure S2) and length fragmentation pattern (electronic supplementary material, figures S3 and S4). We further validated it with PMD tools [12], a statistical tool designed to isolate endogenous from contaminant DNA sequences; the PMD score distribution obtained is shifted toward positive values (electronic supplementary material, figures S5 and S6), which is characteristic of ancient samples. Several precautions were taken to account for the low coverage and the existence of single DNA reads: the ends of the reads were trimmed to eliminate potential post-mortem damage and C–T and G–A substitutions were only considered when they were shared with other rodent species.

We further inferred a time-calibrated Bayesian phylogenetic tree, relying on three secondary calibration points based on the results of Aghová *et al*. [13]. Bayesian inference (BI) was used to estimate the phylogenetic relationships and node ages using the BEAST v. 2.6.5 package [14]. The multiple sequence alignment was built using MAFFT software [15]. We first aligned arvicanthin sequences and then we added to this alignment the sequence of †*C. bravoi* using the –add option in MAFFT. Best fit model of nucleotide substitution for this alignment was elected with jModelTest [16] based on the Bayesian information criterion.

To infer the time-calibrated phylogeny, we used the Bayesian uncorrelated lognormal relaxed clock (ULRC) model implemented in BEAST v. 2 [17]. We used a coalescent model tree prior with a constant population size [18]. We set three palaeontological calibration points at different nodes of the tree: a *Mus*/*Rattus* divergence between 11.6 and 13.8 Ma [13,19], an *Arvicanthis*/*Lemniscomys* divergence between 6.1 and 8.5 Ma [20,21] and a basal node of the Arvicanthini of 8.5–9.2 Ma [13,20].

The Tree Model was set to a birth–death speciation process [22] to account more accurately for extinct and missing lineages. We used BEAST 2 to run 180 million generations of the model to sample trees from the posterior distribution (each 5000 generations). After examining effective sample sizes (ESS) and the traces for posterior, prior and likelihood with the tool Tracer [23], we discarded the first 20% of trees from the analysis (burn-in proportion). Finally, we generated the tree with median age estimates and 95% higher posterior densities (95% HPD bars) using tree annotator tool (distributed with the BEAST v. 2 package). Convergence of runs was assessed by examining the ESS of parameters, using the recommended threshold of 200 [17].

### (d) Evolutionary rates

We calculated evolutionary rates for the body mass (in grams) increase from *A. niloticus* to †*C. bravoi*. Mean body mass for *A. niloticus* (sexes combined) is taken from Monadjem *et al*. [24], while estimated mean body mass for †*C. bravoi* is taken from Moncunill-Solé *et al*. [25] and is based on multiple regressions considering dental, cranial and postcranial measurements.

Evolutionary rates are calculated using the simple classic equation by Haldane [26]:

2.1 $$r=\left(\ln(x1)-\frac{\ln(x2)}{\Delta t}\right),$$

where, *r* is the rate of change (in darwins, d); *x*1 and *x*2 are the initial and final value of the analysed variable, respectively; and Δ*t* is the amount of time elapsed. The calculations are carried for the minimum and maximum divergence dates between *A. niloticus* and †*C. bravoi*. The age of the oldest †*C. bravoi* fossils (17 300 cal BP [4,8]) is taken as the endpoint of the size increase trend. Calculated evolutionary rates are compared to those derived for other mammals (e.g. [27,28]) as well as to those for †*M. insularis*, which is included because it represents another case of murid gigantism in the same archipelago. Body mass for †*M. insularis* is estimated from published cranial and postcranial measurements [29], applying described equations [25]. Estimated mean body mass for †*M. insularis* is compared to that of its closest relatives, *Mus* (*Coelomys*) *pahari* and *Mus* (*Coelomys*) *crociduroides* from Southeastern Asia [30] with calculations formulated for minimum and maximum divergence dates between †*M. insularis* and *Mus* (*Coelomys*) spp. The oldest †*Malpaisomys* fossils have been dated to 32 000 cal BP [30], which is taken as the age for the end of the size increase trend.

## 3. Results and discussion

A total of 1 616 176 nucleotides mapped to *Arvicanthis* nuclear genome, representing only a 0.0006× depth of coverage but proving that genomic retrieval of †*C. bravoi* is a possible—albeit a challenging—task.

**Figure 2.**
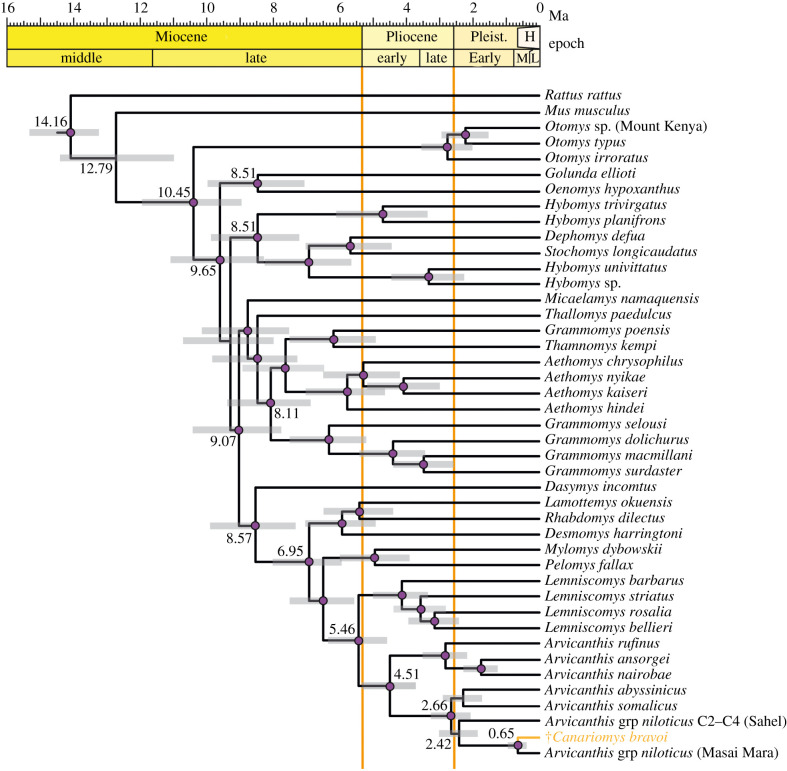
Molecular phylogenetic tree of the Muridae with the mitochondrial DNA data. Median ages are indicated at the nodes while error bars (grey shading) at nodes correspond to the 95% highest posterior density (HPD) intervals of age estimates. Purple circles at nodes indicate posterior probabilities greater than 95%. NCBI codes for each mitogenome can be found at electronic supplementary material, table S1.

A total of 2627 mapped mtDNA nucleotides were aligned to a large dataset of rodent mitogenomes (table S1) and subsequently used for the phylogenetic analysis (table 1). The resulting dated phylogeny confidently places †*C. bravoi* within the *Arvicanthis* genus, in the *A. niloticus* species complex [30] (figure 2); it is closely related to a specimen from Masai Mara (Kenya) and is more distinctly related to a specimen belonging to the C2–C4 lineage that is distributed across the Sahel (both *Arvicanthis* specimens correspond to cryptic species in the *A. niloticus* species complex [31]). This unexpected placement parallels that of *M. insularis*, which was found to cluster between members of the genus *Mus* [32], despite its uncommon dental and skeletal traits. †*Canariomys* and †*Malpaisomys* belong to different murine tribes (Murini and Arvicanthini), thus indicating that their origin must be traced to different ancestors and likely different colonization events (the former being much more recent).

**Table 1.** Mapping statistics of †*C. bravoi* mitochondrial and nuclear DNA reads. CB-4 corresponds to shotgun sequencing and CB-10 to mtDNA capture and sequencing. mtDNA reads were mapped following the procedure described in the Methods section; nuclear reads from CB-4 were mapped against the *Arvicanthis niloticus* nuclear genome (NCBI:txid61156).

specimen (mtDNA)	sequenced read pairs	mapped reads	unique q20 reads	BLAST reads	mapped bases	reference recovered
CB-4	119 102 486	281	104	41	1826	10.78%
CB-10	3 746 833	10 745	55	45	3387	7.21%

specimen (nuclear DNA)	sequenced read pairs	mapped reads	unique reads	unique q20 reads	mapped bases	average coverage
CB-4	119 102 486	111 084	40 434	35 593	1 616 176	0.0006X

The expansion of *Arvicanthis* species through North Africa was heavily influenced by Pleistocene climatic fluctuations [33,34]. When environmental conditions changed and the Sahara region dried up, different *Arvicanthis* populations were cornered in areas of grassland and savannah habitats far apart each other. The current patchy distribution of members of the *A. niloticus* species complex includes the Nile River up to the great African lakes, the whole strip of the Sahel and some isolated populations surviving in Pleistocene refuges such as the Hoggar mountains (southern Algeria) (figure 1*b*). A previous molecular study indicated that the *A. niloticus* species complex likely originated in eastern Africa as early as 2 Ma and differentiated in genetically distinct lineages from east to west as a result of Pleistocene climatic cycles [33].

The divergence time between †*C. bravoi* and its closest *Arvicanthis* relative is estimated at 650 000 years ago (95% HPD intervals: 373 000–944 000 years ago) (figure 2). This interval basically includes the Günz and Mindel glaciations, as well as the Günz–Mindel interglacial. This interglacial appears as the most probable period for the colonization of Tenerife by †*Canariomys* ancestors. Interglacial periods altered the monsoon regime and increased rainfall across Africa. Satellite images of the Draa ancient river bed, which drains the anti-Atlas and flows right in front of the Canary Islands, suggests it must have been a river with a flow of more than 400 m^3^ s^−1^ that period and probably dragged logs and masses of vegetation to the sea on which some †*Canariomys* ancestors might have drifted away.

This relatively recent split date points to a rapid evolutionary process associated with gigantism. Body mass of *A. niloticus* ranges between 89 and 130 g (mean 114 g) [24], whereas the estimated weight of †*C. bravoi* was 1492–1650 g (mean 1571 g) [25], which is almost 14 times heavier (we are assuming that the body size of the *A. niloticus* ancestor that originated the *Canariomys* lineage had the same weight than the extant *A. niloticus*). This size increase is comparable to that of the extinct Sicilian giant dormouse †*Leithia melitensis* (13.5 heavier than its putative most closely related species, the garden dormouse *Eliomys quercinus* [11]) and generally well above values inferred for Pleistocene giant murines from several Mediterranean islands (usually 2–3 times heavier than their mainland ancestors [34,35]). It is also far greater than that calculated for †*Malpaisomys*, which is almost four times heavier (90 g) than its mainland relatives (24 g) [30]. It is difficult to assess when the process of size increase was achieved because the age of the oldest †*C. bravoi* fossils is not well constrained and their earliest date for the start of gigantism is necessarily after the splitting from a mainland *Arvicanthis* lineage. According to our data, the resulting temporal range would suppose a yearly increase of mean body mass of just between 0.0015 g and 0.0023 g (considering maximum and minimum splitting dates, respectively). This corresponds to evolutionary rates (in darwins (d)), ranging from 7.09 d to 2.78 d that are well above those observed for non-insular mammals (usually less than 1 d). By contrast, calculated evolutionary rates for †*Malpaisomys* are in the range of those of mainland mammals (0.22–0.16 d). We must remark that our †*Canariomys* evolutionary rate estimates represents a minimal estimate and assumes a constant rate of change. However, large body size may have not been achieved at a constant rate but soon after colonization and stabilized from then on. This would imply even a faster initial growing-size rate for †*Canariomys*.

However, only the retrieval of additional †*Canariomys* nuclear genome-wide data would further clarify its evolutionary history and would also allow the identification of the genomic regions under selection that might be responsible for the conspicuous physical differences observed between this extinct lineage and its living relatives.

## Data accessibility

Genetic data generated are deposited in the Dryad database (project https://doi.org/10.5061/dryad.9s4mw6mhn).
